# The elicitation of patient preferences for hip replacement surgery: a discrete choice experiment

**DOI:** 10.1186/s12913-025-12393-6

**Published:** 2025-02-18

**Authors:** Stefan Rohrbacher, Martin Emmert

**Affiliations:** 1 Institute for Health Care Management and Health Sciences, University of Bayreuth, Prieserstrasse 2, Bayreuth, 95444 Germany; 2 Bavarian Cancer Registry, Bavarian Health and Food Safety Authority, Meistersingerstrasse 2, Bayreuth, 95444 Germany

**Keywords:** Public reporting, Hospital choice, Patient preferences, Discrete choice experiment, Composite measures, Hospital report cards

## Abstract

**Background:**

The calculation of aggregated composite measures is a widely used approach to reduce the amount of quality-related data on hospital report cards (HRCs). This study aims to elicit patient preferences for hospital choice concerning publicly available hospital quality information for total hip replacement surgery. The results may assist in the development of weighted composite measures for elective hip replacement, which facilitates a conscious selection of the hospital.

**Methods:**

We collect primary survey data on a sample of 364 randomly selected users of the German HRC “Weisse Liste” (WL) (4/5 2023). The key attributes for hospital choice are based on the information provided in WL. We run various model specifications to identify patient preferences, allowing the analysis of unobserved preference heterogeneity.

**Results:**

Our sample consists of 177 respondents (mean age 56.46; 43.5% female). All attributes used are statistically significant for hospital choice ($p<0.01$). Patients consider the “Quality of treatment” (26.95%; level range 1.734) and “Number of cases treated” (24.78%; level range 1.594) to be the most important. In contrast, “EndoCert Certificate” (17.50%; level range 1.126), “Equipment and qualification” (15.83%; level range 1.018), and “Recommendation from other patients” (14.94%; level range 0.960) remain less important. We find no evidence for unobserved heterogeneity regarding the preferences for hospital choice.

**Conclusion:**

Based on our findings, HRC users value publicly available hospital quality information for elective hip replacement differently. These differences should be taken into account when calculating aggregated composite measures. Our results may allow the calculation of a weighted aggregate composite measure from the perspective of HRC users.

**Supplementary Information:**

The online version contains supplementary material available at 10.1186/s12913-025-12393-6.

## Introduction

Total hip replacement (THR) is one of the most frequent surgeries worldwide; approximately one million patients undergo THR surgery annually [1]. In Germany, THR is one of the most common inpatient surgeries, with nearly 250,000 procedures per year. More than 160,000 patients with hip arthrosis undergo primary elective THR annually in approximately 1,250 hospitals [2]. Thereby, the frequency of THR surgeries in Germany differs widely from region to region; Schäfer et al. [3] report a variation factor of 2.8 between federal states, with higher rates in the south and the north-west. They claim that this may be caused – at least to some extent – by “the absence of standardized decision criteria as basis for the indication of THR in a transparent and consistent way”.

The numbers shown above demonstrate that patients in Germany have the option to choose from a large number of hospitals to undergo THR surgery. However, data from the German external hospital quality assurance system indicate that the effectiveness of treatment (i.e. the success of the surgery) varies between hospitals. Therein, for each patient, hospitals must document certain interventions as THR surgery based on a set of in-hospital related quality indicators [2]. The latest German hospital quality report from 2024 lists the share of hospitals with quality deficits ranging between 4.15%, regarding “Prevention of falls measures” (47 out of 1,132 hospitals), and 24.46%, regarding “Indication for hip endoprosthesis or component replacement” (251 out of 1,026 hospitals) [4]. These quality differences are not surprising since other studies also demonstrate hospital-related variations in the quality of THR surgery. For example, a recently published meta-analysis of 44 studies focusing on the impact of hospital volume on the outcomes of THR indicates that low-volume hospitals are associated with higher rates of surgical site infections, 90-day complications, costs, and mortality [1]. Previous evidence from Germany shows lower risk-adjusted in-hospital mortality rates for THR in high-volume hospitals compared to low-volume hospitals (0.10% vs. 0.23%) [5].

Against this backdrop, it seems important for patients to be able to make an informed choice; i.e. to select the best-performing or “right” hospital. Therefore, public reporting aims to support patients and other consumers by providing quality information about health care providers such as hospitals, nursing homes, or practitioners [6]. For this purpose, publicly available Internet rating websites have been developed and implemented in many high-income countries like the United Kingdom, the United States, and Germany [7–9]. For example, the leading German public reporting portal “Weisse Liste” (in English, white list, WL) was jointly initiated by the non-profit foundation “Bertelsmann Stiftung” and the main patients’ and consumers’ associations in 2008 [9]. More recently, the operation of WL ceased in March 2024; however, it will serve as an important element of its successor “Klinik-Atlas” in terms of, e.g. user guidance, selected quality measures, and other aspects [10]. The latter is the first German government-run public reporting website with the intention to provide quality-related information on the hospital level for the public as of May 2024 [11]. So far, there is limited quality information regarding the detailed types of information that will be provided on “Klinik-Atlas” but it is likely to be extended shortly and become similar to WL (e.g. number of cases treated, clinical measures, staff-related information), apart from novel types of information [12].

Public reporting websites should fulfil a number of requirements in order to have an impact on health care delivery [13–16]. They should present the information that consumers value most while keeping the amount of quality information low in order to minimise complexity [17, 18]. As shown in previous literature, calculating composite measures based on consumer preferences is one way to achieve this [18–26].

The objective of our study is to learn more about consumers’ preferences when choosing a hospital for THR surgery on the German public reporting website WL. We focus exclusively on the publicly reported quality information about hospitals regarding elective THR surgery and exclude parameters related to patients as individuals. This may help in the future to calculate transparent weighted composite measures, which may ease patients making a conscious choice. Hence, we address the following three questions:

1. How do patients rate different publicly available hospital quality information regarding THR surgery?
2. What relative importance do patients assign to different types of quality information?
3. Can we identify groups of respondents with similar preferences?

## Methods

### Methodology of the discrete choice experiment

The objective of this study is to elicit patients’ preferences regarding hospital choice for THR surgery through the utilisation of a discrete choice experiment (DCE). Building upon the theoretical work of McFadden [27] and Lancaster [28], a DCE is a method that uses (survey) data on stated preferences based on a set of hypothetical choice scenarios (e.g. hospital choice) to systematically investigate the structure of individuals’ preferences. DCEs allow for reduced complexity for respondents due to the pairwise comparison of alternatives, which is an advantage compared with traditional formats for the elicitation of stated preferences, like rating- and ranking-based methods [29]. In addition, they also facilitate realistic trade-off decisions [30–32]. Therefore, DCEs appear increasingly often in the field of preference research in health care [33].

The design and analysis of the DCE are based on standardised research practices for conducting conjoint analysis developed by the ISPOR Conjoint Analysis Good Research Practices Task Force [34–36]. However, in contrast to previous studies focusing on the relative importance of quality information for hospital choice in general (e.g. [25]), we aim to investigate how patients rate the hospital quality information with respect to THR surgery publicly available on WL and the relative importance patients assign to this information. Based on this, our results might help determine relative weights for publicly reported quality information on WL for the calculation of a composite performance measure. Such weighted composite measures may be integrated in health report services in the future to support patients making a conscious hospital choice. Thus, instead of conducting both a systematic literature review and qualitative research (e.g. semi-structured interviews, focus groups) to identify and choose the most important attributes for patients’ preferences (see, e.g. [25, 29, 37]), we select our attributes in line with the publicly reported quality information provided on WL, one of the major health report cards in Germany at the time of the experiment. We focus on the information items primarily presented to users in the search interface of WL: (1) “Quality of treatment”, which indicates whether a hospital fulfils obligatory quality targets such as, e.g. certain treatment outcomes; (2) “Recommendation from other patients”, which provides information on the experiences of previous patients; (3) “Annual number of cases treated”, which is supposed to reflect the experience of a hospital, based on the assumption that high numbers of cases indicate more in-depth experience with the special treatment; (4) “Equipment and qualification”, which covers the (non-)satisfaction of compulsory quality targets in the provision of medical equipment and the appropriate qualification of medical staff; and (5) “EndoCert Certificate”, which indicates possible certifications that a hospital may have received from the “EndoCert Certification System”, an independent initiative for quality auditing for endoprosthetic care founded by the German Society for Orthopaedics and Orthopaedic Surgery [38]. We focus exclusively on criteria on hospital quality and refrain from regarding individual patient-related features as e.g. travelling distance which depend on each patient’s situation and willingness to share such information. Moreover, due to transparency and simplicity the composite measure should only build upon primary information in order to be useful for each patient. Each attribute contains three levels (see Table 1). We explain and illustrate each attribute to participants using simple examples and basic facts (for a detailed list of underlying information for each attribute as used on WL, please refer to Table A.1 of the Supplementary Material). We employ effect coding on all attributes (e.g. [36, 39]). Effect coding enables us to construct estimates and standard errors of all levels against their deviation from each attribute’s mean. Hence, both estimates and standard errors are stable and independent of an arbitrary benchmark level.

**Table 1:** Summary of attributes and levels used in the discrete choice experiment

Attribute	*Description* and levels given in the questionnaire
Quality of treatment	*Total hip replacements with fulfilled treatment criteria*
	Quality targets reached
	No assessment available / results not (yet) available
	Quality targets not reached
Recommendation from other patients	*Share of positive recommendations of other patients*
	Recommended by 85% (above average)
	Recommended by 80% (average)
	Recommended by 76% (below average)
Number of cases treated	*The annual number of total elective hip replacement surgeries in a hospital*
	230 patients (above average)
	148 patients (average)
	84 patients (below average)
Equipment and qualification	*Hospitals with fulfilled criteria on medical equipment and staff qualification*
	Quality targets reached
	No assessment available / results not (yet) available
	Quality targets not reached
EndoCert Certificate	*Hospitals which meet requirements of the EndoCert Certification System*
	Certified EndoProstheticsCentre of Maximum Care (EPCmax)
	Certified EndoProstheticsCentre (EPC)
	No certificate

### Survey design

There are four parts to the survey. In the first part, we collect information about the participants’ motivation to use WL, their expectations, and their previous experience with HRCs. In the second part, respondents are presented with information on all five attributes and their levels and are asked to rate the attributes on a scale of 1–5 (1 = not at all important; 5 = very important) and to rank them against each other. The third section contains ten DCE choice tasks in which respondents are asked to choose between two hypothetical hospitals. The final section collects socio-demographic and health-related information on participants for subgroup analyses. Participants were also given the opportunity to provide feedback on the survey and to enter a prize drawing for 10 online vouchers worth EUR 50 each. Before the survey was launched, the questionnaire was anonymously piloted for clarity and comprehensibility by 20 people and modified accordingly.

### Experimental design

We designed the survey using Sawtooth Software (Lighthouse Studio Version 9.14.0) as a full profile design, i.e. each choice set includes all five attributes. We generated the final set using the balanced overlap method, which permits the estimation of both main and interaction effects with standard errors below 0.05 and 0.1 and the highest D-efficiency score [40–42]. The choice tasks are forced-choice tasks, i.e. respondents have to choose one of two hypothetical hospitals by making trade-offs between attributes and levels [25]. With this approach, the experiment provides a setting that is close to reality, as comparable trade-off decisions are part of daily life [32]. We administered the survey as an onsite-based survey on the German HRC Weisse Liste (WL). Over a 2-month period (April and May 2023), we invited all users of WL who either searched for information on THR surgery or performed hospital comparisons on THR surgery to participate in our study. Participants were free to take part and could terminate the survey at any time. Please refer to the supplementary material for an English translation of the questionnaire.

### Sample size

Our study design consists of ten choice tasks per respondent, including two alternatives (i.e. hospitals) per choice task and a maximum number of three levels across all attributes. As suggested by Orme [43], the design specifications require a minimum of participants according to $N\ge \frac{500l}{ta}$, where *N* is the number of respondents, *t* the number of tasks, *a* the number of alternatives and *l* the maximum of levels. In our setting with $\{t,a,l\}=\{10,2,3\}$, we derive a minimum of 75 participants. However, since this number indicates only the lower bound limit for main effect estimation, we require at least 150 respondents, as recommended for achieving statistical robustness [44, 45].

### Data analyses

For data analyses, we use R Statistical Software (Version 4.2.2; R Foundation for Statistical Computing, Vienna, Austria) and the corresponding packages “mlogit” [46] and “gmnl” [47]. As a starting point, we use the standard multinomial logit model (MNL), as initially developed by McFadden [27]. MNL is attractive because of its simplicity in terms of estimation and interpretation. However, it relies on the rather restrictive assumptions that preferences are homogeneous across individuals and that error terms are independently and identically distributed, both of which may conceal unobserved heterogeneity. We relax both assumptions with various alternative model specifications to test for unobserved heterogeneity. We consider this important because undetected heterogeneity may affect the estimation results and thus the appropriate weights of the included attributes, which may then be used for the calculation of a composite measure. Especially in the case of undetected group heterogeneity, we would need to adjust the composite measure for appropriate subgroups.

We use the random parameter model (RPL), as proposed by McFadden and Train [48], in order to relax the assumption of taste homogeneity (while maintaining independent and identically distributed errors). This model extends MNL by using a continuously distributed random parameter for each individual. In addition, we retreat from independently and identically distributed errors (while maintaining homogeneous preferences) and assume that idiosyncratic errors are not identical but individually scaled, as suggested by Bhat [49] and Fiebig et al. [50] (S-MNL). Similar to RPL, S-MNL also captures unobserved heterogeneity. However, its advantage over RPL is its more parsimonious description, which allows for more efficient estimation. Next, we use the general MNL model (G-MNL) introduced by Fiebig et al. [50], which combines the characteristics of RPL and S-MNL. As pointed out by Fiebig et al. [50], G-MNL puts more weight on randomness in the tails compared to RPL due to the inclusion of scaled error terms. Therefore, G-MNL captures unobserved heterogeneity better than RPL, which focuses more on the centre of the distribution. Besides assuming a continuous distribution of preference heterogeneity, as in the models above, we also estimate standard latent class models (LC) [51] and latent class models enhanced by random parameters (MM-MNL) [52]. These model types assume a discrete distribution of heterogeneity. In consequence, the unobserved heterogeneity clusters in groups rather than at the individual level, as in RPL or G-MNL. In the case of MM-MNL, both types of heterogeneity are combined so that individual unobserved heterogeneity can occur within clustered groups. In order to identify the best-fitting model, the Bayesian information criterion (BIC) and conditional Akaike information criterion (CAIC) are used. We refrain from using the Akaike information criterion (AIC) since it is known to be insufficiently restrictive, especially in multi-class models, favouring too many groups, whereas the BIC and CAIC perform well (see, e.g. [53]).

## Results

### Sample characteristics

In total, 5,042 consumers of WL opened the survey link provided on WL. Of these, 4,678 stopped answering the survey directly after the short introduction page. Thus, 364 respondents returned the questionnaire. Clearly, the drop-out rate of 96.5% is rather high. However, our main target is to derive possibly accurate weights for a composite measure and therefore not necessarily to draw a representative sample which is likely to contain observations from users who do not have clear preferences or are unwilling to participate, as these we consider the primary source of noisy data. In the light of our objectives, we assume the drop-out rate not problematic. The following analysis reports only on the 177 respondents who fully completed the DCE part of the survey and provided consistent responses (48.63% completion rate). Table 2 summarises the key characteristics of the sample. The median age of all respondents is 58 years, slightly more than half of all respondents are female (54.80%), and 68.93% stated (technical) university entrance qualification as their highest educational level. In addition, 53.56% of all participants report their health status to be good or better, and 54.80% claim that they suffer from a chronic condition. Finally, 58.76% of respondents state that they have used hospital report cards during the last 12 months, and more than eight out of ten surveyed respondents (84.75%) state that they perceived big differences in the quality of care between hospitals. Please note that all respondents acknowledge differences in hospital quality which is in line with the studies and reports mentioned in the introduction [1, 4, 5].

**Table 2:** Key characteristics of participants

Participants (*N* = 177)	*n*	%
**Age**		
39 years and younger	22	12.43%
40 to 49 years	24	13.56%
50 to 59 years	46	25.99%
60 to 69 years	58	32.77%
70 years and older	24	15.25%
missing	3	1.69%
**Gender**		
Female	97	54.80%
Male	77	43.50%
Diverse	3	1.69%
**Educational Attainment**		
(Technical) University entrance qualification	122	68.93%
Intermediate secondary school	42	23.73%
Secondary general school or less	12	6.78%
Missing	1	0.56%
**Health Status**		
Good or better	95	53.67%
Satisfactory	57	32.20%
Bad or worse	20	11.30%
Missing	5	2.82%
**Chronic conditions**		
Any chronic condition	97	54.80%
No chronic condition	75	42.37%
Missing	5	2.82%
**Health insurance**		
Public health insurance	128	72.32%
Private health insurance	48	27.12%
Missing	1	0.56%
**Perceived differences in hospital quality**		
Big differences	150	84.75%
Small differences	27	15.25%
No differences	0	0%
**Experience with hospital report cards**		
Yes	104	58.76%
No	73	41.24%

### Descriptive rating and ranking results

The results regarding the importance of the five presented quality information items for the hospital choice for THR surgery (on a scale of 1–5, with $1=$ not at all important and $5=$ extremely important) show that the “Quality of treatment” is rated as the most relevant ($4.76\pm 0.56$), followed by “Equipment and qualification” ($4.73\pm 0.49$) and “Number of cases treated” ($4.62\pm 0.70$) (see Table 3). In contrast, holding an “EndoCert Certificate” ($4.15\pm 0.85$) and “Recommendation from other patients” ($3.96\pm 0.84$) seem to be less important. The ranking results for the single most relevant information item reveal “Quality of treatment” (47.46%) and “Number of cases treated” (35.03%) as the most relevant, while “EndoCert Certificate” (9.60%), “Equipment and qualification” (6.21%), and “Recommendation from other patients” (1.69%) are stated less frequently.

**Table 3:** Importance of different information items for the treatment choice

Attributes (*N* = 177)	Rated importance of different information items		Ranked single most important information items	
	Mean	SD	*n*	%
Quality of treatment	4.76	0.56	84	47.46%
Recommendation from other patients	3.96	0.84	3	1.69%
Number of cases treated	4.62	0.70	62	35.03%
Equipment and qualification	4.73	0.49	11	6.21%
EndoCert Certificate	4.15	0.85	17	9.60%

### Model choice

For single-class models, we find that the MNL model represents the data best with respect to all consulted information criteria. In addition, relative estimation results vary only marginally across single-class models (cf. Table A.3 of Supplementary Material). The same picture emerges with respect to multi-class models. Regarding the number of classes for the multi-class models, the BIC and CAIC point at using a single class only (cf. Table A.4 of Supplementary Material). Moreover, we find only a little variation of relative estimations even for multi-class models. For both LC and MM-MNL with two groups, for example, one group evaluates the number of cases treated higher and the other prefers recommendations, while all other attributes remain roughly as before (cf. Table A.5 of Supplementary Material). However, using two groups does not improve the data match sufficiently compared to the (single-class) MNL model such that the BIC and CAIC reject these models. Expanding to even more classes provides no further insights. This indicates that unobserved heterogeneity, either as idiosyncratic continuous error or in the form of clustered groups, is negligible in our sample. We conclude that additional model assumptions provide no meaningful information. Therefore, we hereinafter restrict our discussion to the MNL model.

### Findings from the DCE

**Fig. 1:**
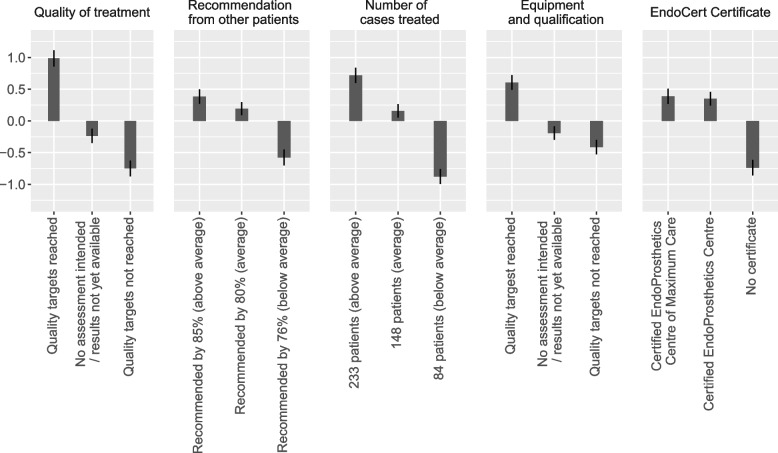
Graphical display of level estimates with 95%CI of attributes for hospital choice

Table 4 summarises the estimation results of the MNL model. Since we employ effect coding, all estimates are with respect to the (hypothetical) mean of the corresponding attribute, implying that the coefficient values within an attribute add up to zero. As shown, all estimates are highly significant. Consequently, all attributes are highly relevant for the hospital choice of the respondents. Overall, consumers prefer hospitals that achieve the quality targets for the treatment quality, as well as for equipment and qualification, and have above-average numbers for the cases treated and patient recommendations, as well as being certified as an EndoProstheticsCentre of Maximum Care (EPCmax). This pattern also emerges from Fig. 1, which depicts the corresponding level estimates.

**Table 4:** Estimated parameters of the multinomial logit model (MNL)

Attributes and levels	Coeff.	SE	95% CI		*p*-value	Difference highest to lowest level
**Quality of treatment**						1.734
Quality targets reached	0.985	0.064	0.858	1.112	<0.001	
No assessment intended	−0.236	0.057	−0.349	−0.124	<0.001	
Quality targets not reached	−0.749	0.063	−0.873	−0.624	<0.001	
**Recommendation from other patients**						0.960
Recommended by 85% (above average)	0.384	0.057	0.271	0.498	<0.001	
Recommended by 80% (average)	0.192	0.052	0.089	0.294	<0.001	
Recommended by 76% (below average)	−0.576	0.063	−0.700	−0.452	<0.001	
**Number of cases treated**						1.594
230 patients (above average)	0.718	0.061	0.598	0.837	<0.001	
148 patients (average)	0.158	0.053	0.054	0.263	0.003	
84 patients (below average)	−0.876	0.060	−0.994	−0.758	<0.001	
**Equipment and qualification**						1.018
Quality targets reached	0.605	0.058	0.490	0.720	<0.001	
No assessment intended	−0.192	0.054	−0.298	−0.086	<0.001	
Quality targets not reached	−0.413	0.058	−0.527	−0.300	<0.001	
**EndoCert Certificate**						1.126
Certified EPCmax	0.388	0.060	0.269	0.507	<0.001	
Certified EPC	0.350	0.054	0.243	0.456	<0.001	
No certificate	−0.738	0.062	−0.860	−0.616	<0.001	

*SE* standard error, *CI* confidence interval

**Fig. 2:**
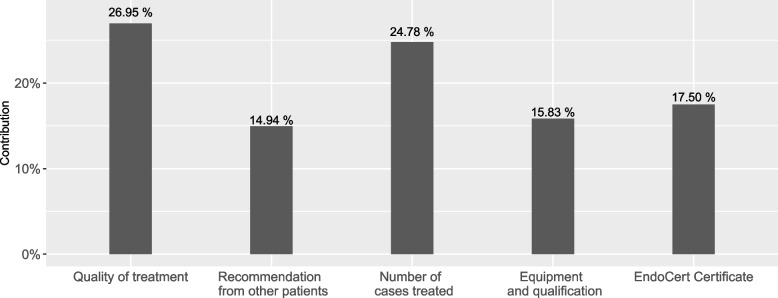
Graphical display of mean relative importance of attributes for hospital choice

Figure 2 illustrates the mean relative importance of attributes for hospital choice. Thereby, the range of coefficient values within an attribute reflects the importance of an attribute for the hospital choice. We compute the relative importance of each attribute using each attribute’s coefficient range (i.e. the difference between the coefficients of the highest and lowest level of each attribute), expressed as a share of the total range across attributes. As shown, patients consider the quality of treatment as the most important (26.96%; level range of 1.734), followed by the annual number of cases treated (24.78%; level range of 1.594). In contrast, holding an EndoCert Certificate (17.51%; level range of 1.126), reaching quality targets with respect to equipment and qualification (15.83%; level range of 1.018), and recommendations from other patients (14.93%; level range of 0.960) are less important.

## Discussion

The objective of this study is to elicit patient preferences concerning publicly available hospital quality information for elective hip replacement surgery by conducting an onsite-survey on the German HRC Weisse Liste (WL). The findings may support us in developing weighted composite measures from the consumers’ perspective. We can draw several conclusions from our results. First, we can see that the DCE-based findings appear broadly consistent with the results of the rating- and ranking-based findings. This applies to both the order and relative importance of all quality information items. As shown, “Quality of treatment” is the most important information in all three analyses. For example, the DCE shows a relative importance of 26.95%, whereas 47.46% of the respondents ranked it in first place; it was also rated highest in terms of the mean importance, with a score of 4.76 (on a 1–5 scale with 1 = not at all important and 5 = extremely important). In addition, “Recommendations from other patients” appears to be the least important information in all three approaches. Here, the DCE shows a relative importance of 14.94%, whereas 1.69% of the respondents ranked it in first place; it was also rated lowest in terms of the mean importance, with a score of 3.96. We find slightly different results for the remaining three attributes, which, however, are not contradictory overall. For example, “Number of cases treated” is placed second with respect to the relative importance of all five attributes. Here, we compute a relative weight of 24.78% based on the DCE, and 35.03% of the respondents ranked it in first place as the single most important information item; however, the mean importance is rated 4.62 and thus is slightly lower than the mean importance of “Equipment and qualification”. Nevertheless, considering the standard deviations and the small differences of the means, the higher mean of “Equipment and qualification” from the rating-based approach may not be meaningful compared with the results from the ranking-based approach and the DCE-based findings.

Second, the attributes for hospital choice for THR employed in this study – or joint replacement in general – have been utilised in other DCE studies, and thus appear to be of significant importance in general. For example, several studies include “Quality of treatment” [54–56]. The “Number of cases treated” is also identified as a factor influencing hospital choice [25, 55–57]. In other studies, “Equipment and qualification” [58], “EndoCert Certificate” [25, 57], and “Recommendations from other patients” [57, 59] are applied in a hypothetical hospital choice scenario. Subsequently, our study may incorporate the most important attributes for hospital choice, despite the absence of qualitative research to identify and select the most pertinent attributes for patient preferences in general [25, 29, 37]. However, we select our attributes in accordance with the publicly reported quality information provided on WL (see above).

Furthermore, we find evidence for the importance of the attributes of this study from more general approaches. Quality improvements of hospitals are associated with high case volume [1, 60, 61], certification [62, 63] or better equipment and experience of medical staff [64]. Additionally, all but “Recommendation from other patients” are among the top ten relevant information items for hospital choice among THR patients in Germany [65]. However, we would like to emphasise that all the studies mentioned above include a variety of attributes that may not be present in our setting or may have been used differently. For example, “Quality of treatment” might also contain adverse medical outcomes such as complication rates or readmission rates. In addition to this, “Equipment and qualification” might also cover the conduct of medical staff.

Third, despite the differences in the general setting and combination of attributes, which do not allow for the direct comparison of findings, we may compare the relative importance of our attributes with findings from other research. In general, our results seem to be similar to those from other studies. “Quality of treatment” shows high importance in other studies [25, 54–56, 59]. For example, Emmert et al. [25] compare the preferences of both patients and referring physicians regarding hospital quality information for THR surgery. They demonstrate that traditional quality measures are rated most important (e.g. postoperative complication rates, one-year revision surgery). In the case of “Number of cases treated”, we find more heterogeneous results. While Emmert et al. [25] and Kuklinski et al. [57] report “Number of cases treated” to be a highly important attribute, Groenewoud et al. [55] and Damman et al. [56] determine a medium relevance yet still find it to be more important than patient recommendations or the qualifications of staff, respectively. With respect to the remaining attributes, our results demonstrate their lower importance for hospital choice. For example, “Equipment and qualification” [56, 58], “EndoCert Certificate” [25], and “Recommendation from other patients” [59] appear to be less important. Moreover, Kuklinski et al. [57] find for knee replacement that patient recommendation is more important than certification with case volume being the most important quality indicator. Finally, it should be mentioned that our study is similar to the research of Emmert et al. [25], who analyse the preferences of hip replacement patients after surgery. Given that we recruited participants for the survey during their hospital search on WL, we expect that our results are more likely to reflect the preference picture before THR treatment. Both studies demonstrate that patients evaluate “Number of cases treated” as relatively important and holding an “EndoCert Certificate” as less relevant.

Fourth, we find that the order of levels is consistent and as expected for all attributes. However, we find that the preference for EPCmax certificates is only 10.9% higher than that for EndoprostheticsCentre certificates (EPC). Similarly, Emmert et al. [25] report relatively small differences between preferences for EPCmax and EPC certificates among both patients and physicians. We suspect that this may be due to patients not being aware of the differences between the two certificates. The certificates are issued by EndoCert Limited, which is a subsidiary of the German Society for Orthopaedics and Orthopaedic Surgery [66]. The certification process requires the fulfilment of comprehensive quality objectives regarding structure, processes, and outcomes. Depending on which criteria are met, a hospital will receive one or the other certificate. For example, an EPC must treat at least 100 patients per year, whereas an EPCmax must treat at least 200 patients per year [38]. Alternatively, the added value of higher certificate types may be of limited relevance. This would imply that patients may know the differences but may not care about the type of certificate a hospital holds as long as it provides one. However, at this point we can only speculate about the reasons for this finding. Since our findings are consistent with Emmert et al. [25], we recommend further research to explore the reason for this result in more detail.

Fifth, we cannot detect various respondent groups with similar preferences. All considered multi-class models are inferior to the MNL model as the best model with respect to BIC and CAIC. The differences seem minor or implausible and do not make a case for dividing into sub-groups (cf. Tables A.4 and A.5 of Supplementary Material).

Finally, as suggested by previous research, the results of our study on patient preferences can be used to develop weighted composite measures from the consumer perspective [22, 25]. Such a composite measure would aggregate existing information into one summary score, thereby improving the usability of a health report card like WL by reducing the complexity of the information provided. In this context, Schlesinger et al. [17] or Emmert et al. [67] show that reducing the complexity of report cards increases the quality of users’ hospital choices. Based on our findings, it may be possible to calculate the weights for a patient-centred composite measure that is publicly reported on WL. The next step would be to convert real hospital quality results into hospital-related composite measures. The preference for each hospital can then be estimated based on the sum of part-worth utilities for the selected level of all attributes [68]. For example, a hospital that achieves quality targets regarding the treatment quality (coef. 0.985) and equipment and qualification (coef. 0.605), with above-average recommendations from other patients (coef. 0.384), above-average case numbers (coef. 0.718), and certification as an Endoprosthetics Centre of Maxmimum Care (coef. 0.388), would have an overall score of 3.080. Comparing this value for each hospital against the overall score for all hospitals, we can group hospitals into several performance groups [25]. In this context, it seems highly relevant that our different models do not detect unobserved preference heterogeneity in our sample. This implies that it would be sufficient to compute one single weighted composite measure that corresponds to the preferences of all users. Otherwise, it would be appropriate to take into account specific individual or group characteristics.

## Limitations

Our findings should be considered in light of some limitations. First, since we focus on German data from a German population subgroup (i.e. users of the German hospital report card WL), our findings and conclusions might be of limited importance for other countries. However, we think that our methodological approach and results may be of interest for countries with public reporting initiatives (e.g. the United States or United Kingdom). Second, the preferences of respondents derived from hypothetical hospital scenarios might differ from the actual search and selection behaviour in real life when people are confronted with similar decisions [59]. Third, the choice of attributes is based on a pre-existing selection of variables provided on WL during the time of the experiment and not on qualitative pre-study research as recommended (e.g. [29, 37]). Therefore, it is important to mention that other potentially relevant attributes are not considered in our study. We cannot exclude the possibility that integrating other information may lead to different findings. However, the selection of quality information on WL is based on publicly available quality measures from the patient’s perspective; a similar approach was published recently by other authors [59]. Moreover, the chosen attributes are similar to those utilised by Emmert et al. [25], who derive the attributes by means of comprehensive literature research and qualitative research. In both studies, “Number of cases treated” and “EndoCert Certificate” appear, while our “Quality of treatment” and “Equipment and qualification” comprise their “Postoperative complication rate”, “Confirmed diagnosis rate”, “Mobility at hospital discharge”, and “Prevention of fall measures”. Only our attribute “Recommendation from other patients” is entirely novel. Fourth, for “Quality of treatment” and “Equipment and qualification” we model the level types as quality targets reached/not reached. This may lead to overestimating the relative importance of the two attributes since respondents do not need to wager for relative differences as e.g. with level types modelled as mean share of quality targets reached and relative deviations above and below. Yet, even if we overestimate these two, our results for relative importance are very likely to remain qualitatively robust with “Quality of treatment” and “Number of cases treated” being notably more important than the other attributes. Fifth, our results refer to patients for elective hip replacement, i.e. to treatments that focus on improving the quality of life. As shown by Kuklinski et al. [57], patients value quality information differently depending whether the treatment is life-saving or improves quality of life. Hence, our results may not be transferable to other indications as e.g. cancer or stroke where the primary goal of treatment is saving life. Furthermore, they may only add to a weighted composite measure for elective THR or similar indications where treatment is meant to improve the quality of life. Sixth, we need to emphasise that the external validity of our study is limited. Compared to patients of elective THR in 2023, our sample contains slightly too many of age below 50 years, slightly too few of age above 70 years and slightly too many male [69]. In comparison to the general population of Germany, our sample contains higher educational attainment [70]. These differences may be explained partially by regular web-users in Germany, who are younger, more male and better educated [67] as well as by the intertemporal correlation between year of birth and education attainment [70]. Compared to WL users in general, our sample is slightly older, less female and better educated [67]. Taking into account that the major activities of WL next to THR are total knee replacement where patient structure is similar to THR [69] and breast cancer with patients being more female and younger, differences regarding age and sex may narrow down with respect to THR patients on WL. However, our main target is to derive weights for a composite measure. This implies a trade-off between including a representative sample versus only those who see a benefit in participating, i.e., those who are aware of their preferences and eagerly return to use the composite measure. Therefore, the remaining deviations in representativeness seem tolerable. Finally, we cannot entirely exclude sample biases. However, we tried to mitigate the potential risk of biased data by collecting a sufficiently representative sample of adequate size, by providing anonymity for respondents to increase honesty and response quality as well as clear and neutral questions for easy understanding [71].

## Conclusion

This study provides new insights into the preference patterns of HRC users for hospital characteristics for patients undergoing elective THR surgery in Germany. Our results show that patients consider “Quality of treatment” and “Number of cases treated” as highly important. In contrast, “EndoCert Certificate”, “Equipment and qualification”, and “Recommendations from other patients” seem to be less important. We do not detect any meaningful heterogeneity in the preferences of all respondents. Based on our findings, the computation of a weighted composite measure may be the next step.

## Supplementary Information

Supplementary Material 1.

## Data Availability

Data are available on request from the corresponding author (stefan.rohrbacher@uni-bayreuth.de).
