# Supplementary material for: The elicitation of patient preferences for hip replacement surgery: a discrete choice experiment
Source: BMC Health Serv Res. 2025 Feb 18;25:268. doi: 10.1186/s12913-025-12393-6 (PMC11834257; doi:10.1186/s12913-025-12393-6)
Supplement: Supplementary file 1 — Supplementary Material 1. [file 12913_2025_12393_MOESM1_ESM.zip › rohrbacher_emmert_dce_wl_supplementary_material.pdf]

A Supplementary Material

**Quality of treatment**	1. Indication for first elective implantation of endoprosthesis<br>2. Preoperative length of stay<br>3. Fall prevention<br>4. General complications of elective hip endoprosthesis implantation<br>5. General complications of endoprosthetic treatment of a femoral fracture close to the hip joint<br>6. Specific complications of endoprosthetic treatment of a femoral fracture close to the hip joint<br>7. Specific complications of elective hip endoprosthesis implantation<br>8. Inability to walk upon discharge<br>9. Hospital mortality<br>10. Hip endoprosthesis or component replacement during the course of the procedure
**Recommendations from other patients**	Recommendations stated by previous patients w.r.t. the hospital, collected by collaborating public health insurances AOK, Barmer and KKH:<br>mean and standard deviations of positive recommendations
**Number of cases treated**	Taken from mandatory official hospital reports:<br>mean and standard deviations of reported case volume
**Equipment and qualification**	Medical qualification:<br>1. General surgery<br>2. Orthopaedics and trauma surgery<br>3. Special orthopaedic surgery<br>4. Special trauma surgery<br>Specialised therapeutic staff:<br>5. Masseurs and medical bath attendants<br>6. Orthopaedic mechanics, bandage and shoemakers<br>7. Physiotherapists<br>Medical offers of the specialist departments:<br>8. Joint replacement procedures and endoprosthetics (trauma and reconstructive surgery)<br>9. Endoprosthetics (orthopaedics)<br>10. Examination and treatment of injuries to the hip and thigh<br>11. Examination and treatment of joint diseases<br>12. Surgeries of rheumatic diseases
**EndoCert Certificate**	Certificates issued by EndoCert:<br>1. EndoProstheticsCentre of Maximum Care (EPCmax)<br>2. EndoProstheticsCentre (EPC)

Table A.1: List of information comprised in the attributes

Which of the following hospitals would you choose?		
	**Hospital 1**	**Hospital 2**
**Quality of treatment**	Quality targets reached	No assessment available / results not (yet) available
**Recommendation from other patients**	76% (below average)	80% (average)
**Number of cases treated**	230 patients (above average)	148 patients (average)
**Equipment and qualification**	No assessment available / results not (yet) available	Quality targets not reached
**Endocert Certificate**	Certified EndoProstheticsCentre of Maximum Care (EPCmax)	Certified EndoProtheticsCentre (EPC)

Table A.2: Example choice task

	MNL	S-MNL	G-MNL		RPL	
	Coefficients		Coefficients	SD	Coefficients	SD
**Quality of treatment**						
Quality targets reached	0.985*** (0.064)	1.147*** (0.175)	1.461*** (0.171)	0.208 (0.168)	1.500** (0.490)	0.523 (0.416)
No assessment intended/results not (yet) available	−0.246*** (0.057)	−0.288*** (0.081)	−0.361*** (0.086)	−0.268 (0.293)	−0.337* (0.143)	−1.444 (0.953)
Quality targets not reached	−0.749*** (0.063)	−0.860*** (0.130)	−1.099*** (0.137)	0.060 (0.242)	−1.164** (0.396)	0.921 (0.661)
**Recommendation from other patients**						
recommended by 85% (above average)	0.384*** (0.057)	0.423*** (0.076)	0.564*** (0.096)	0.027 (0.238)	0.570** (0.203)	0.427 (0.586)
recommended by 80% (average)	0.192*** (0.052)	0.226*** (0.694)	0.294*** (0.076)	−0.186 (0.295)	0.311* (0.127)	−0.645 (0.711)
recommended by 76% (below average)	−0.576*** (0.063)	−0.649*** (0.103)	−0.858*** (0.123)	0.159 (0.173)	−0.881** (0.293)	0.218 (0.469)
**Number of cases treated**						
230 patients (above average)	0.718*** (0.061)	0.818*** (0.119)	1.059*** (0.131)	0.352** (0.124)	1.117** (0.379)	0.634 (0.402)
148 patients (average)	0.158*** (0.053)	0.178*** (0.063)	0.223** (0.082)	−0.713*** (0.196)	0.242* (0.120)	−1.167** (0.421)
84 patients (below average)	−0.876*** (0.060)	−0.995*** (0.138)	−1.281*** (0.145)	0.361*** (0.109)	−1.359** (0.459)	0.533 (0.450)
**Equipment and qualification**						
Quality targets reached	0.605*** (0.058)	0.715*** (0.124)	0.903*** (0.122)	0.286* (0.125)	0.920** (0.313)	0.073 (0.395)
No assessment intended/results not (yet) available	−0.192*** (0.054)	−0.218*** (0.067)	−0.251** (0.079)	−0.377 (0.300)	−0.308* (0.131)	−0.079 (0.545)
Quality targets not reached	−0.413*** (0.058)	−0.497*** (0.102)	−0.652*** (0.103)	0.091 (0.280)	−0.612** (0.218)	0.006 (0.326)
**Endocert Certficate**						
Certified EndoProstheticsCentre of Maximum Care	0.388*** (0.060)	0.463*** (0.103)	0.644*** (0.115)	0.193 (0.158)	0.568** (0.198)	0.587 (0.457)
Certified EndoProstheticsCentre	0.350*** (0.054)	0.397*** (0.075)	0.441*** (0.081)	−0.389 (0.222)	0.503** (0.167)	−0.784 (0.669)
No certificate	−0.738*** (0.062)	−0.860*** (0.140)	−1.085*** (0.138)	0.196 (0.149)	−1.071** (0.330)	0.197 (0.399)
τ	–	0.471* (0.225)	0.615*** (0.108)		–	
γ	–	–	11.609 (297.171)		–	
Log Likelihood	−825.70	−824.75	−807.02		−821.04	
BIC	1726.18	1731.78	1778.57		1791.65	
CAIC	1736.18	1742.78	1800.57		1811.65	
Random draws	–	2000	2000		2000	

Note: *p<0.05; **p<0.01; ***p<0.001

Table A.3: Results of various regression models

	LC		MM-MNL	
Number of classes	BIC	CAIC	BIC	CAIC
1	1726.18	1736.18	1791.65	1811.65
2	1731.25	1752.25	1855.92	1896.92
3	1755.62	1787.62	1956.64	2018.64
4	1796.30	1839.30	2080.44	2163.44
5	1854.12	1908.12	2172.32	2276.32

Table A.4: Information criteria statistics for model evaluation - selecting number of classes in multi-class models

	MNL	LC		MM-MNL			
	Coefficients	LC 1	LC 2	Coefficients Class 1	SD Class 1	Coefficients Class 2	SD Class 2
**Quality of treatment**							
Quality targets reached	0.985*** (0.064)	1.305*** (0.194)	1.078*** (0.098)	1.632*** (0.0.279)	0.922*** (0.241)	1.207*** (0.119)	0.005 (0.251)
No assessment intended/results not (yet) available	−0.246*** (0.057)	−0.680*** (0.190)	−0.136 (0.089)	−0.827*** (0.238)	−1.419*** (0.315)	−0.142 (0.095)	−0.019 (0.340)
Quality targets not reached	−0.749*** (0.063)	−0.625*** (0.177)	−0.941*** (0.092)	−0.806*** (0.219)	0.497 (0.313)	−1.064*** (0.276)	0.013 (0.232)
**Recommendation from other patients**							
recommended by 85% (above average)	0.384*** (0.057)	0.168 (0.149)	0.553*** (0.093)	0.126 (0.171)	0.054 (0.265)	0.718*** (0.114)	0.010 (0.162)
recommended by 80% (average)	0.192*** (0.052)	0.374** (0.125)	0.171* (0.082)	0.493*** (0.144)	−0.131 (0.610)	0.166 (0.102)	−0.408 (0.214)
recommended by 76% (below average)	−0.576*** (0.063)	−0.543*** (0.163)	−0.724*** (0.094)	−0.620** (0.189)	0.077 (0.571)	−0.884*** (0.129)	0.397** (0.139)
**Number of cases treated**							
230 patients (above average)	0.718*** (0.061)	1.820*** (0.258)	0.221 (0.129)	2.223*** (0.340)	0.022 (0.445)	0.207 (0.121)	0.013 (0.178)
148 patients (average)	0.158*** (0.053)	0.114 (0.122)	0.214*** (0.075)	0.227 (0.154)	−0.384 (0.545)	0.221* (0.091)	−0.097 (0.414)
84 patients (below average)	−0.876*** (0.060)	−1.934*** (0.259)	−0.435*** (0.125)	−2.450*** (0.386)	0.362 (0.289)	−0.427*** (0.120)	0.084 (0.366)
**Equipment and qualification**							
Quality targets reached	0.605*** (0.058)	0.761*** (0.131)	0.633*** (0.087)	0.948*** (0.173)	0.010 (0.234)	0.674*** (0.101)	0.014 (0.300)
No assessment intended/results not (yet) available	−0.192*** (0.054)	−0.250 (0.134)	−0.196** (0.078)	−0.240 (0.149)	−0.016 (0.369)	−0.224* (0.100)	−0.395 (0.332)
Quality targets not reached	−0.413*** (0.058)	−0.511*** (0.142)	−0.438*** (0.092)	−0.709*** (0.168)	0.007 (0.276)	−0.450*** (0.112)	0.381** (0.143)
**Endocert Certficate**							
Certified EndoProstheticsCentre of Maximum Care	0.388*** (0.060)	0.663*** (0.155)	0.293* (0.115)	0.967*** (0.212)	0.033 (0.703)	0.258* (0.118)	0.212 (0.174)
Certified EndoProstheticsCentre	0.350*** (0.054)	0.496*** (0.131)	0.346*** (0.076)	0.645*** (0.182)	−0.655 (0.713)	0.374*** (0.093)	−0.269 (0.529)
No certificate	−0.738*** (0.062)	−1.159*** (0.157)	−0.639** (0.115)	−1.613*** (0.276)	0.622** (0.225)	−0.632*** (0.116)	0.057 (0.509)
intercept class 2	–	–	0.317** (0.119)	–	–	0.139 (0.086)	–
Log Likelihood	−825.7	−787.1		−774.65			
BIC	1726.18	1731.25		1855.92			
CAIC	1736.18	1752.25		1896.92			
Random draws	–	–		2000			

Note: *p<0.05; **p<0.01; ***p<0.001

Table A.5: Results of regressions with two latent classes
