# Supplementary material for: The elicitation of patient preferences for hip replacement surgery: a discrete choice experiment
Source: BMC Health Serv Res. 2025 Feb 18;25:268. doi: 10.1186/s12913-025-12393-6 (PMC11834257; doi:10.1186/s12913-025-12393-6)
Supplement: Supplementary file 1 — Supplementary Material 1. [file 12913_2025_12393_MOESM1_ESM.zip › rohrbacher_emmert_dce_wl_survey_en.pdf]

Beginning

Survey on the procedure for choosing a clinic

Dear participants ,

As part of a research project at the University of Bayreuth, we are investigating which criteria patients use to select a hospital for an operation to insert an artificial hip or knee joint (TEP operation). Their answers will help us to better understand the needs of patients when choosing a hospital. The findings can then be addressed to politicians, health insurance companies and doctors.

The questionnaire consists of four parts:

1. General experience and expectations of a clinic guide

2. Importance of the various quality information when choosing a clinic

3. Choice between different hospitals

4. General information

The survey takes about 10 minutes. It is important for the success of this study that all questions are answered completely and truthfully. The survey is anonymous and cannot be traced back to you. The data collected will be treated in strict confidence and analysed exclusively for scientific purposes.

Competition

All participants aged 18 and over will be entered into a prize draw to win 20 gift vouchers worth 25 euros each, which can be redeemed in over 500 participating shops! You can leave your e-mail address at the end of the survey. The e-mail addresses will only be collected for this purpose and will of course be deleted afterwards.[1]

Thank you very much in advance for your participation!

Prof. Dr Martin Emmert

Prof. Dr Florian Meier

Pascal Lehmann

University of Bayreuth

[1] The winners will receive an e-mail with a voucher code that they can redeem at wunschgutschein.de. All participating shops can be viewed at wunschgutschein.de. The prize will be drawn on 01.06.2023. The winners will be chosen at random from all participants and informed of the prize by e-mail. You can revoke your consent to the competition at any time by sending a message. Legal recourse is excluded.

Group of p e o p l e

Which of the following groups of people do you belong to?

[Please tick the appropriate box]

Group of people=1

○ Patient

Group of people=2

○ Relatives/friends of a patient

Group of people=3

○ Medical staff (e.g. referring doctor)

Group of people=4

Person group_4_other

○ Other: ______

0% 100%

UNIVERSITÄT BAYREUTH

Expectati ons

What expectations do you have of the Weisse Liste when searching for a hospital?

[Multiple answers are possible]

Expectations_1 ☐ I would like to find the right hospital

Expectations_2 ☐ I would like to determine the quality of a particular hospital

Expectations_3 ☐ I would like to compare potential hospitals

Expectations_4 ☐ I am looking for general information about a hospital (e.g. address)

Expectations_5 ☐ I would like to prepare for a consultation with my doctor

Expectations_6 ☐ I am generally interested in the content of the Weisse Liste

Expectations_7 ☐ Expectations_7_other Other: ____________

0% 100%

UNIVERSITÄT BAYREUTH

Existingdecision

Have you already decided on a specific clinic for your upcoming treatment?

[Please tick the appropriate box]

ExistingDecision=1

◯ **Yes**, the choice of clinic has already been made

ExistingDecision=2

◯ **No**, the choice of clinic has not yet been made

ExistingDecision=3 ExistingDecision_3_other

◯ Miscellaneous [ ]

0% 100%

Differences in quality

Do you think that there are differences in the quality of treatment in different hospitals when inserting an artificial hip or knee joint?

[Please tick the appropriate box]

Differences in quality=1 ◯ Yes, there are **big** differences

Differences in quality=2 ◯ Yes, there are **slight** differences

Differences in quality=3 ◯ No, there are **no** differences

0% 100%

Necessity

How necessary do you think internet portals, such as the Weisse Liste, are for presenting the quality of hospitals?

[Please tick the appropriate box]

Not necessary at all	Little necessary	Partly Partly	Necessary	Very necessary
Necessity_r1=1	Necessity_r1=2	Necessity_r1=3	Necessity_r1=4	Necessity_r1=5
○	○	○	○	○

0% 100%

UNIVERSITÄT BAYREUTH

AwarenessWL

Have you ever compared clinics on the Weisse Liste in the past twelve months?

AwarenessWL=1 Yes

AwarenessWL=2 No

AwarenessOther

Have you ever compared clinics on other clinic guides on the Internet (e.g. Klinikbewertungen.de, TK- Klinikführer) in the past twelve months?

AwarenessOther=1 Yes

AwarenessOther=2 No

0% 100%

UNIVERSITÄT BAYREUTH

Introduction

Please put yourself in the following situation:

Your orthopaedic surgeon has diagnosed you as needing an artificial hip or knee joint. Unfortunately, all other attempts at treatment have not been successful. He/she names some clinics in your region where you could have the operation performed; however, he/she does not have a clear recommendation. You decide to find out about the clinics yourself. You have recently heard about so-called**clinic guides on the Internet**, which provide quality information about clinics, including those for the insertion of an artificial hip or knee joint. You decide to take a closer look at these clinic guides and familiarise yourself with both the quality information available and the clinics in question.

For the following questions, we would like to know what significance the **quality information** shown there has for your choice of clinic?

0% 100%

UNIVERSITÄT BAYREUTH

SubjectiveTreatment quality

Importance of the quality information shown on clinic guides (1/5)

Treatment quality

For certain treatments or operations, hospitals are legally obliged to document and report data on the quality of treatment. This includes information on the outcome of treatment, the number of deaths and complications that have occurred. This information can be used to determine whether a hospital has fulfilled the statutory quality requirements.

There are three possibilities:
- *Quality targets reached*
- *Quality targets not reached*
- *No assessment intended / results not (yet) available*

How important is the information on *"treatment quality"* to you when choosing a clinic?

[Please tick the appropriate box]

Not important at all	Less important	Partly important	Rather important	Very important
SubjectiveTreatmentQuality_r1=1 ○	SubjectiveTreatmentQuality_r1=2 ○	SubjectiveTreatmentQuality_r1=3 ○	SubjectiveTreatmentQuality_r1=4 ○	SubjectiveTreatmentQuality_r1=5 ○

0% 100%

UNIVERSITÄT BAYREUTH

SubjectiveRecomendation

Importance of the quality information shown on clinic guides (2/5)

Recommendation by patients

The "Recommendation by patients" is based on a patient survey conducted by The health insurers AOK, BARMER and KKH. It represents the proportion of patients who Would recommend the hospital to their friends.

There are three possibilities:

- *Recoomended by 85% (above average)*
- *Recommended by 80% (average)*
- *Recommended by 76% (below average)*

How important is the information on *"patient recommendation"* to you when choosing a clinic?

[Please tick the appropriate box]

Not important at all	Less important	Partly important	Rather important	Very important
SubjectiveRecommendation_r1=1	SubjectiveRecoomendation_r1=2	SubjectiveRecoomendation_r1=3	SubjectiveRecommendation_r1=4	SubjectiveRecommendation_r1=5
○	○	○	○	○

0% 100%

UNIVERSITÄT BAYREUTH

SubjectiveNumber of cases

Importance of the quality information shown on clinic guides (3/5)

Number of cases treated

This number describes how often a clinic has implanted an artificial hip or knee joint in a year. The average number of cases in Germany is currently148 patients operated on per year.

There are three possibilities:
- *230 patients (above average)*
- *148 patients (average)*
- *84 patients (below average)*

How important is the information on the *"number of cases treated"* for you when choosing a clinic?

[Please tick the appropriate box]

Not important at all	Less important	Partly important	Rather important	Very important
SubjectiveCase_number_r1=1	Subjective_number_of_cases_r1=2	Subjective_number_of_cases_r1=3	Subjective_number_of_cases_r1=4	Subjective_number_of_cases_r1=5
○	○	○	○	○

0% 100%

UNIVERSITÄT BAYREUTH

SubjectiveAusandQualif

Importance of the quality information shown on clinic guides (4/5)

Equipment and qualification

The hospital must be as suitable as possible for the patient's treatment requirements. The necessary equiment (e.g. required devices) and appropriate qualifications of the medical staff (e.g. training) should be available for the insertion of the artificial hip or knee Joint. This information indicates whether a clinic has fulfilled thes quality requirements

There are three possibilities:

- *Quality targets reached*
- *Quality targest not reached*
- *No assessment available / results not (yet) available*

How important is the information on *"equipment and qualification"* to you when choosing a clinic?

[Please tick the appropriate box]

Not important at all	Less important	Partly important	Rather important	Very important
SubjectiveAusandQualifi_r1=1	SubjectiveAusandQualifi_r1=2	SubjectiveAusandQualifi_r1=3	SubjectiveAusandQualifi_r1=4	SubjectiveAusandQualifi_r1=5
○	○	○	○	○

0% 100%

UNIVERSITÄT BAYREUTH

SubjectiveCertificate

Importance of the quality information shown on clinic guides (5/5)

EndoCert Certificate

Orthopaedic clinics can b e certified as an EndoProsthetics Centre (EPC) or as an EndoProsthetics Centre of Maximum Care (EPCmax). For this purpose, experts examine the structures, processes and medical results of a clinic. If certain requirements are met (e.g. at least 100 hip and/or knee operations per year for EPCs or 200 hip and/or knee operations for EPCmax), the clinics are certified as EPCs or EPCmax.

There are options here:
- Certified EndoProsthetics Cenre (EPC
- Certificied EndoProsthetics Centre of Maximum Care (EPCmax)
- *No* certificate

How important is information on *"EndoCert Certificate" to you when choosing a clinic?*

[Please tick the appropriate box]

Not important at all	Less important	Partly important	Rather important	Very important
SubjectiveCertificate_r1=1	SubjectiveCertificate_r1=2	SubjectiveCertificate_r1=3	SubjectiveCertificate_r1=4	SubjectiveCertificate_r1=5
○	○	○	○	○

0% 100%

UNIVERSITÄT BAYREUTH

RankingQi

Please now categorise the different quality information according to your perceived importance.

[Please rank the different quality information from top (most important) to bottom (least important) by clicking and dragging the information on the left side]

Quality information	
RankingQi_4 Equipment and qualification	Most important for me
RankingQi_5 EndoCert Certificate	
RankingQi_1 Quality of treatment	
RankingQi_3 Number of cases treated	
RankingQi_2 Recommendation from other patients	
	Most insignificant for me

0% 100%

UNIVERSITÄT BAYREUTH

IntroductionDCE

Note for the following section of the survey!

In the following, we present two clinics - in the style of clinic guides on the Internet - and would like to know which of the two clinics you would choose. The results of the quality information just shown are presented for each clinic.

Note: In reality, your choice of clinic will probably include other criteria (e.g. distance from your home, personal experience with a clinic in the past, personal contacts). Please assume that this information about the clinics can be categorised as the same or similar.

0% 100%

UNIVERSITÄT BAYREUTH

DCComposite_Random1

Which of the following two clinics would you choose?

(1 of 10)

	Clinic 1	Clinic 2
**Number of cases treated**	84 (below average)	148 (average)
**Recommendation from other patients**	85% (above average)	76% (below average)
**Quality of treatment**	No assessment avalaible / results Not (yet) available	Quality targets not reached
**Equipment and qualification**	Quality targets reached	Quality targets not reached
**EndoCert certificates**	Certified Endorosthetics Centre of Maximum Care (EPCmax)	No certificate
	DCComposite_Random1 **I would choose this clinic**	DCComposite_Random1 **I would choose This clinic**

0% 100%

UNIVERSITÄT BAYREUTH

DCComposite_Random1

Which of the following two clinics would you choose?

(2 of 10)

	Clinic 1	Clinic 2
**Number of cases treated**	230 (above average)	230 (above average)
**Recommendation from other patients**	85% (above average)	80% (average)
**Quality of treatment**	Quality targets not reached	Quality targets reached
**Equipment and qualification**	Quality targets reached	Quality targets reached
**EndoCert certificates**	Certified Endoprosthetics Centre of Maximum Care (EPCmax)	Certified Endoprosthetics Centre (EPC)
	DCComposite_Random2 **I would choose this clinic**	DCComposite_Random2 **I would choose This clinic**

0% 100%

UNIVERSITÄT BAYREUTH

DCComposite_Random1

Which of the following two clinics would you choose?

(3 of 10)

	Clinic 1	Clinic 2
**Number of cases treated**	148 (average)	84 (below average)
**Recommendation from other patients**	85% (above average)	76% (below-average)
**Quality of treatment**	No assessment avalaible / results Not (yet) available	Quality targets reached
**Equipment and qualification**	No assessment avalaible / results Not (yet) available	Quality targets not reached
**EndoCert certificates**	Certified Endorosthetics Centre of Maximum Care (EPCmax)	No certificate
	DCComposite_Random3 **I would choose this clinic**	DCComposite_Random3 **I would choose This clinic**

0% 100%

DCComposite_Random1

Which of the following two clinics would you choose?

(4 of 10)

	Clinic 1	Clinic 2
**Number of cases treated**	230 (above average)	148 (average)
**Recommendation from other patients**	76% (below average)	80% (average)
**Quality of treatment**	Quality targets reached	No assessment avalaible / results Not (yet) available
**Equipment and qualification**	No assessment avalaible / results Not (yet) available	Quality targets not reached
**EndoCert certificates**	Certified Endorosthetics Centre of Maximum Care (EPCmax)	Certified Endorosthetics Centre (EPC)
	DCComposite_Random4 **I would choose this clinic**	DCComposite_Random4 **I would choose This clinic**

0% 100%

DCComposite_Random1

Which of the following two clinics would you choose?

(5 of 10)

	Clinic 1	Clinic 2
**Number of cases treated**	148 (average)	84 (below average)
**Recommendation from other patients**	80% (average)	85% (above average)
**Quality of treatment**	Quality targets not reached	Quality targets not reached
**Equipment and qualification**	No assessment avalaible / results Not (yet) available	Quality targets reached
**EndoCert certificates**	Certified Endorosthetics Centre of Maximum Care (EPCmax)	No certificate
	DCComposite_Random5 **I would choose this clinic**	DCComposite_Random5 **I would choose This clinic**

0% 100%

UNIVERSITÄT BAYREUTH

DCComposite_Random1

Which of the following two clinics would you choose?

(6 of 10)

	Clinic 1	Clinic 2
**Number of cases treated**	230 (above average)	84 (below average)
**Recommendation from other patients**	76% (below average)	80% (average)
**Quality of treatment**	Quality targets reached	No assessment avalaible / results Not (yet) available
**Equipment and qualification**	Quality targets not reached	Quality targets reached
**EndoCert certificates**	No certificate	Certified Endorosthetics Centre (EPC)
	DCComposite_Random6 **I would choose this clinic**	DCComposite_Random6 **I would choose This clinic**

0% 100%

DCComposite_Random1

Which of the following two clinics would you choose?

(7 of 10)

	Clinic 1	Clinic 2
**Number of cases treated**	230 (above average)	84 (below average)
**Recommendation from other patients**	80% (average)	76% (below average)
**Quality of treatment**	Quality targets not reached	Quality targets reached
**Equipment and qualification**	No assessment avalaible / results Not (yet) available	Quality targets not reached
**EndoCert certificates**	No certificate	Certified Endorosthetics Centre (EPC)
	DCComposite_Random7 **I would choose this clinic**	DCComposite_Random7 **I would choose This clinic**

0% 100%

UNIVERSITÄT BAYREUTH

DCComposite_Random1

Which of the following two clinics would you choose?

(8 of 10)

	Clinic 1	Clinic 2
**Number of cases treated**	230 (above average)	148 (average)
**Recommendation from other patients**	76% (below average)	80% (average)
**Quality of treatment**	No assessment avalaible / results Not (yet) available	Quality targets not reached
**Equipment and qualification**	No assessment avalaible / results Not (yet) available	Quality targets reached
**EndoCert certificates**	Certified Endorosthetics Centre of Maximum Care (EPCmax)	No certificate
	DCComposite_Random8 **I would choose this clinic**	DCComposite_Random8 **I would choose This clinic**

0% 100%

UNIVERSITÄT BAYREUTH

DCComposite_Random1

Which of the following two clinics would you choose?

(9 of 10)

	Clinic 1	Clinic 2
**Number of cases treated**	84 (below average)	148 (average)
**Recommendation from other patients**	85% (above average)	85% (above average)
**Quality of treatment**	Quality targest reached	Quality targets reached
**Equipment and qualification**	Quality targets reached	Quality targets not reached
**EndoCert certificates**	Certified Endorosthetics Centre of Maximum Care (EPCmax)	Certified Endorosthetics Centre of Maximum Care (EPCmax)
	DCComposite_Random9 **I would choose this clinic**	DCComposite_Random9 **I would choose This clinic**

0% 100%

UNIVERSITÄT BAYREUTH

DCComposite_Random1

Which of the following two clinics would you choose?

(10 of 10)

	Clinic 1	Clinic 2
**Number of cases treated**	230 (above average)	148 (average)
**Recommendation from other patients**	85% (above average)	76% (below average)
**Quality of treatment**	No assessment avalaible / results Not (yet) available	No assessment avalaible / results Not (yet) available
**Equipment and qualification**	Quality targets not reached	Quality targets reached
**EndoCert certificates**	Certified Endorosthetics Centre (EPC)	Certified Endorosthetics Centre (EPC)
	DCComposite_Random10 **I would choose this clinic**	DCComposite_Random10 **I would choose This clinic**

0% 100%

EndIntroduction

Last part of the survey

You have now almost reached the end of the survey. Finally, we would like to ask you for some demographic information. This information **does not** allow **any** conclusions to be drawn about you personally, but is important for the scientific analysis.

You can then leave your e-mail address to take part in the prize draw for 20 vouchers worth €25 each.

0% 100%

UNIVERSITÄT BAYREUTH

Gender

Please enter your gender.

Gender=1 Male

Gender=2 Female

Gender=3 Miscellaneous

A g e

Please enter your age.

years

0% 100%

Education

Please enter your highest educational qualification.

Education=1 ◯ Still in school education

Education=2 ◯ Secondary school leaving certificate

Education=3 ◯ Secondary school leaving certificate (Realschulabschluss and comparable school-leaving qualifications)

Education=4 ◯ University of Applied Sciences or university entrance qualification

Education=5 ◯ Without a general school leaving certificate

Marital status

Please state your marital status.

Marital status=1 ◯ Divorced

Marital status=2 ◯ Single

Marital status=3 ◯ Married Living separately

Marital status=4 ◯ Married and living together

Marital status=5 ◯ Registered civil partnership living separately

Marital status=6 ◯ Registered civil partnership living together

Marital status=7 ◯ Widowed

Health insurance

Please indicate your health insurance status.

Health insurance=1 ◯ Statutory health insurance

Health insurance=2 ◯ Private health insurance

0%

100%

Chronic illness

Apart from the illness that led to the implantation of the artificial hip or knee joint, do you have a chronic illness or a long-term health problem?

ChronicDisease=1
○ Yes

Chronicdisease=2
○ No

State of health

What is your general state of health?

Health status=1
○ Very good

Health status=2
○ Good

Health status=3
○ Mediocre

Health status=4
○ Bad

Health status=5
○ Very bad

0% 100%

Suggestions

Do you have any further suggestions or would you like to tell us about additional points that we have not asked about?

0% 100%

EmailAddress

Raffle of 20 gift vouchers worth €25 each

Finally, please leave us your e-mail address if you would like to take part in the prize draw for the 20 vouchers worth €25 each. This gives us the opportunity to contact you if you win a voucher. Your data will only be stored until the winners are drawn and only collected for this purpose. Afterwards, the data will of course be deleted. Your data will not be passed on to third parties.

E-mail address:

0% 100%

UNIVERSITÄT BAYREUTH

End

You have made it and have now reached the end of the survey!
Thank you very much for taking part in our survey and for your support.

If you have any questions, please do not hesitate to contact us at any time. Your study team of the research project at the University of Bayreuth.

Contact information:
Pascal Lehmann
Chair of Medical Management and Health Sciences University of Bayreuth
Prieserstrasse 2
95444 Bayreuth

pascal.lehmann@uni-bayreuth.de

0% 100%
